# Supplementary material for: Defect-Rich Monolayer MoS2 as a Universally Enhanced Substrate for Surface-Enhanced Raman Scattering
Source: Nanomaterials (Basel). 2022 Mar 8;12(6):896. doi: 10.3390/nano12060896 (PMC8953205; doi:10.3390/nano12060896)
Supplement: Supplementary file 1 [file nanomaterials-12-00896-s001.zip › nanomaterials-1615311-supplementary.pdf]

Supporting Information

Defect-Rich Monolayer MoS_2 as a Universally Enhanced Substrate for Surface-Enhanced Raman Scattering

Shiyu Sun [1,†], Jingying Zheng [2,†], Ruihao Sun [1], Dan Wang [1], Guanliang Sun [1], Xingshuang Zhang [1], Hongyu Gong [1], Yong Li [1], Meng Gao [1], Dongwei Li [1,*], Guanchen Xu [1,*] and Xiu Liang [1,*]

[1] Key Laboratory for High Strength Lightweight Metallic Materials of Shandong Province (HM), Advanced Materials Institute, Qilu University of Technology (Shandong Academy of Sciences), Jinan 250014, China; sysun0313@163.com (S.S.); srh1645@163.com (R.S.); Wangdan1910@163.com (D.W.); 13793994223@163.com (G.S.); xszhang@qlu.edu.cn (X.Z.); hygong@sdas.org (H.G.); yongli@sdas.org (Y.L.); mgao@sdas.org (M.G.)

[2] College of Materials Science and Engineering, Fuzhou University, Fuzhou 350108, China; jyzheng@fzu.edu.cn

* Correspondence: dwli@sdas.org (D.L.); gcxu@sdas.org (G.X.); xliang@sdas.org (X.L.)

† These authors contributed equally to this work.

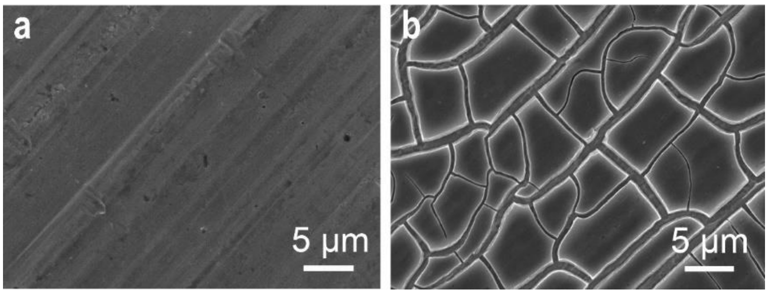

Figure S1. (**a**, **b**) SEM image of the untreated and electroplated surface of Mo foil.

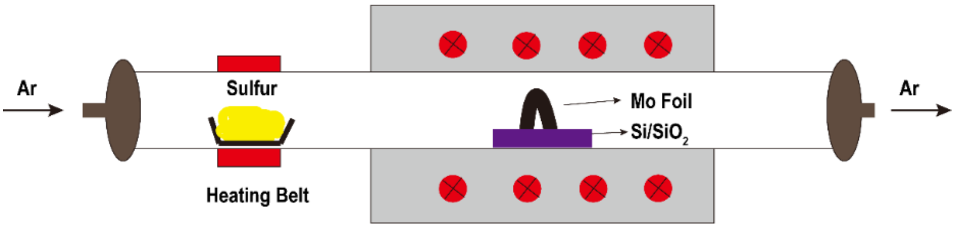

Figure S2. Schematic illustration of CVD system growing monolayer MoS_2.

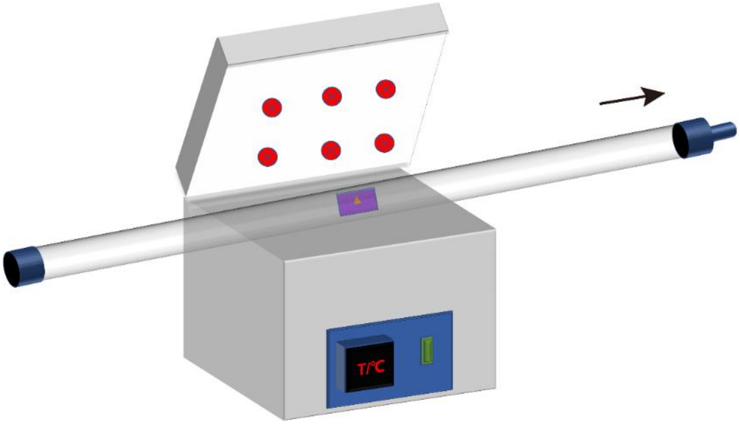

Figure S3. Schematic illustration of the etching process of monolayer MoS_2. Etching of monolayer MoS_2 is performed at temperatures below the growth temperature. The MoS_2 grown on the SiO_2/Si substrate is placed in the center of the glass tube, which is sealed at one end and connected to a vacuum oil pump at the other end.

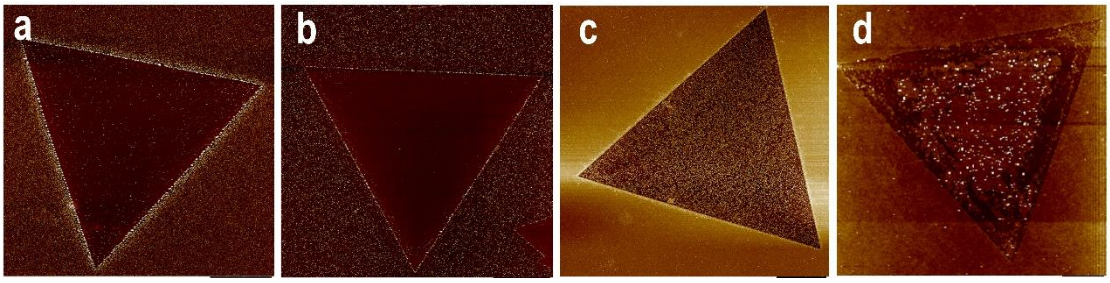

Figure S4. AFM image of (**a**) monolayer MoS_2, (**b**) mild etching monolayer MoS_2, (**c**) moderate etching monolayer of MoS_2, (**d**) severe etching monolayer of MoS_2.

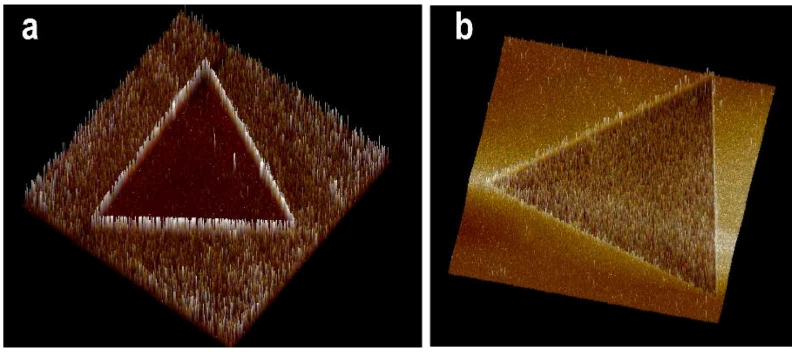

Figure S5. 3D AFM image of (**a**) monolayer MoS_2 and (**b**) moderate etching monolayer MoS_2.

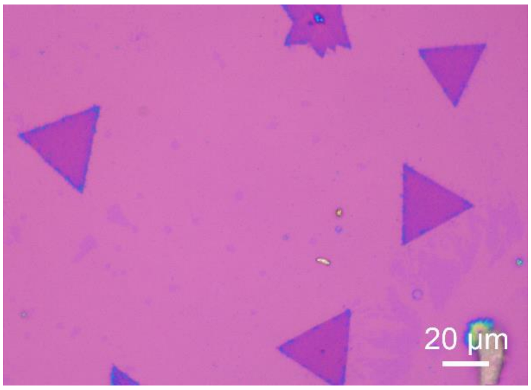

Figure S6. Optical photograph of dye molecules added dropwise on unetched monolayer MoS_2.

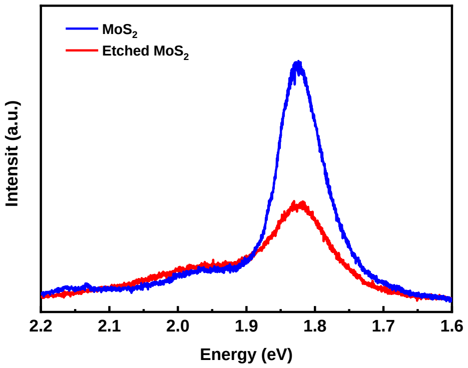

Figure S7. PL spectra of monolayer MoS_2 and etched monolayer MoS_2.

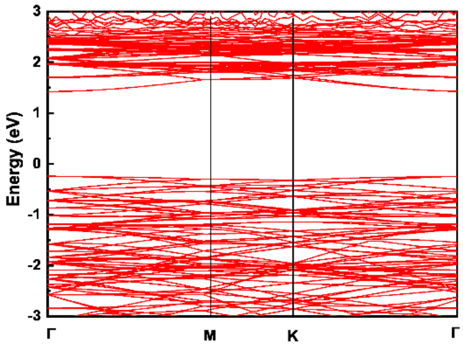

Figure S8. The calculated band structures of MoS_2 take Fermi level as reference.

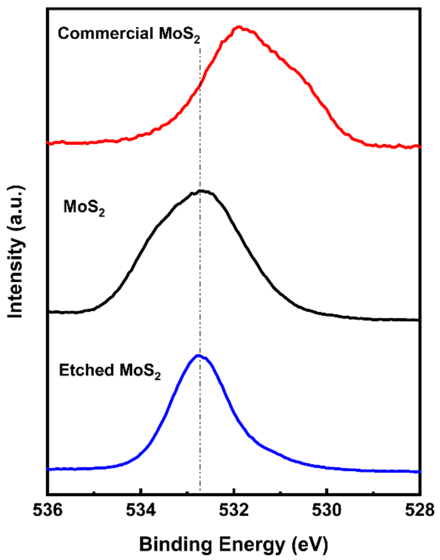

Figure S9. O 1s XPS spectrum of commercial MoS_2 powder, CVD grown monolayer MoS_2, and etched monolayer MoS_2.

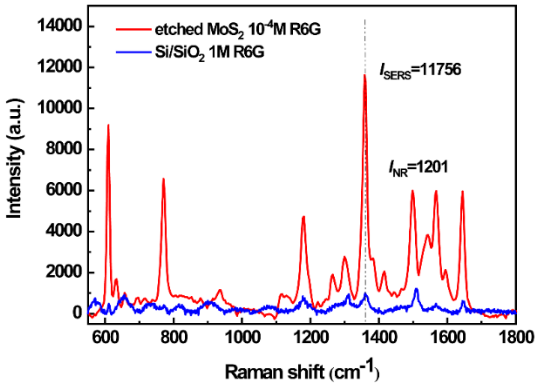

Figure S10. The enhancement factor (EF) of etched MoS_2 overlay 10^{-4}M R6G. Under the same test conditions as "Materials and Methods", we tested the Raman spectra of 10^{-4} M R6G on etched MoS_2 substrates and the Raman spectra of 1 M R6G on SiO_2/Si substrates. The final enhancement factor was obtained 10^5 by selecting the data where 1360 cm^{-1} was located using the calculation formula.

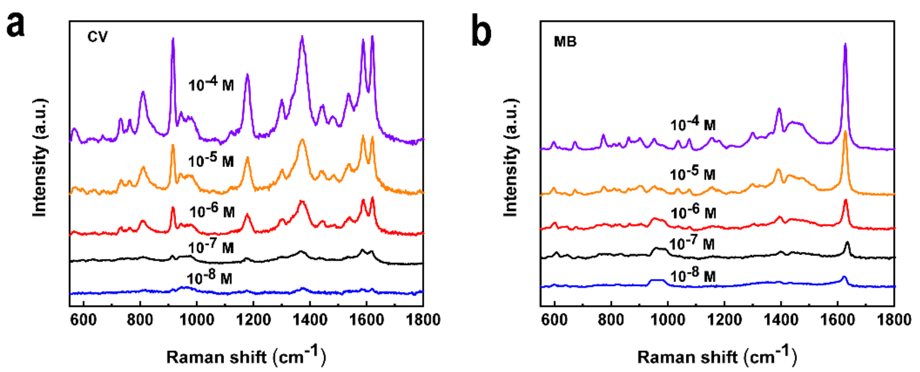

Figure S11. SERS measurements of (**a**) crystal violet (CV), and (**b**) methylene blue (MB) molecules probes on etched monolayer MoS_2 substrates.

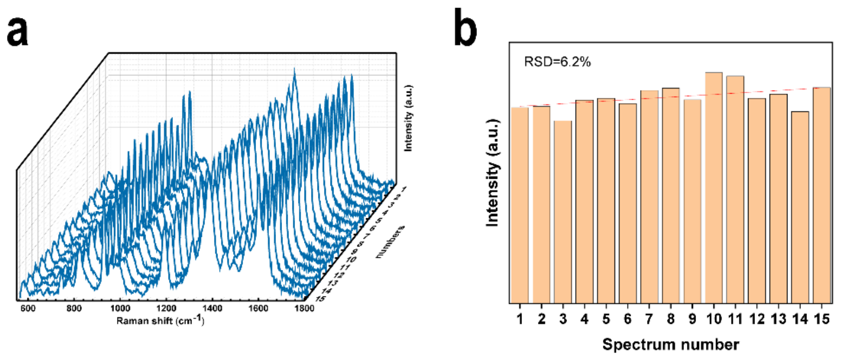

Figure S12. (**a**) SERS spectra of CV (10^{-5} M) coated on etched MoS_2 taken from 15 randomly chosen spots. (**b**) Histogram distribution of the Raman intensities at 1620 cm^{-1}.

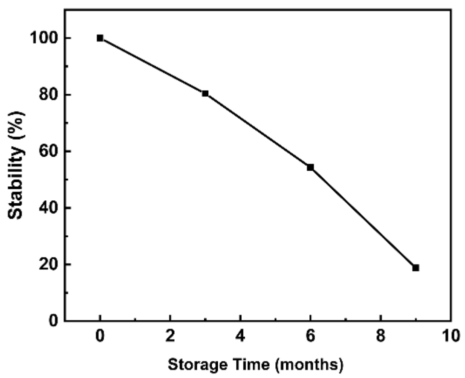

Figure S13. The stability ratio of 10^{-5} M R6G coated on etched MoS_2.

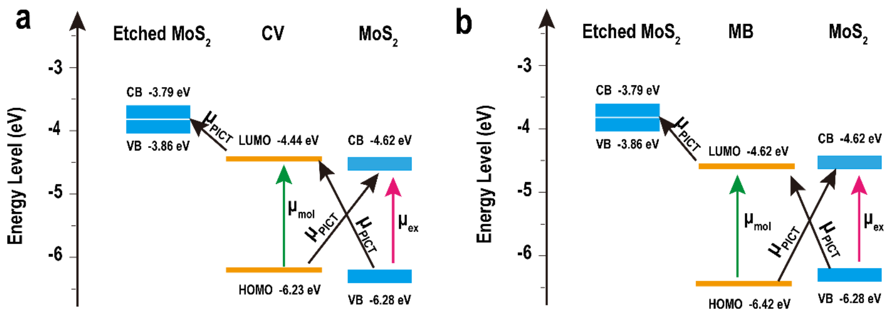

Figure S14. Energy level diagram and charge transfer transitions in the diagram comparing the charge-transfer pathways in (**a**) CV/etched MoS_2 and (**b**) MB/etched MoS_2.

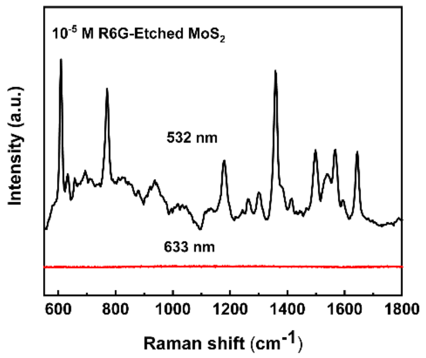

Figure S15. The Raman signals of 10^{-5} M R6G coated on etched MoS_2 were irradiated at 532 nm and 633 nm, respectively.

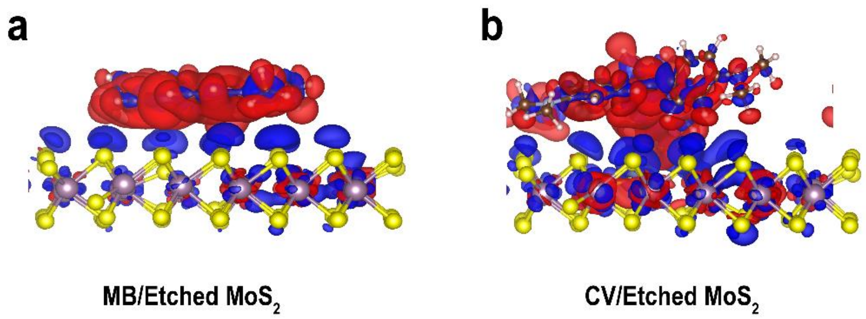

Figure S16. Side views of the electron density difference isosurface for MB (**a**), and CV (**b**) molecule absorbed on etched monolayer MoS_2.
